# Advanced Oxidation Processes Coupled with Nanomaterials for Water Treatment

**DOI:** 10.3390/nano11082045

**Published:** 2021-08-11

**Authors:** Inês M. F. Cardoso, Rita M. F. Cardoso, Joaquim C. G. Esteves da Silva

**Affiliations:** Chemistry Research Unit (CIQUP), DGAOT, Faculty of Sciences of University of Porto (FCUP), Rua do Campo Alegre 697, 4169-007 Porto, Portugal; up201704720@edu.fc.up.pt (I.M.F.C.); up201704723@edu.fc.up.pt (R.M.F.C.)

**Keywords:** AOPs, water treatment, nanomaterials, UV treatments, ozone, hydrogen peroxide, persulfate, chlorine, monochloramine, photocatalysis

## Abstract

Water quality management will be a priority issue in the near future. Indeed, due to scarcity and/or contamination of the water, regulatory frameworks will be increasingly strict to reduce environmental impacts of wastewater and to allow water to be reused. Moreover, drinking water quality standards must be improved in order to account for the emerging pollutants that are being detected in tap water. These tasks can only be achieved if new improved and sustainable water treatment technologies are developed. Nanomaterials are improving the ongoing research on advanced oxidation processes (AOPs). This work reviews the most important AOPs, namely: persulfate, chlorine and NH_2_Cl based processes, UV/H_2_O_2_, Fenton processes, ozone, and heterogeneous photocatalytic processes. A critical review of the current coupling of nanomaterials to some of these AOPs is presented. Besides the active role of the nanomaterials in the degradation of water contaminants/pollutants in the AOPs, the relevance of their adsorbent/absorbent function in these processes is also discussed.

## 1. Introduction

Drinking water scarcity is one of the most problematic crises facing the world today; despite water being one of the most abundant resources in the world, less than 1% is considered clean water available for human consumption [1]. With increasing industrialization, water contamination becomes increasingly evident, essentially due to emerging pollutants such as pharmaceuticals, personal care products and endocrine disrupting compounds [2]. Most conventional water treatment methods do not effectively degrade or remove recalcitrant contaminants or are unable to remove enough to meet increasingly stringent water quality standards, thus indicating the need for alternatives. Additionally, due to the ongoing climate change, there will be an increasing demand for water quality management strategies. In recent decades, research has focused on the development of advanced oxidation processes (AOPs) for water treatment [3].

AOPs are water treatment processes that are promising for the degradation of persistent or toxic organic pollutants, as well as compounds refractory to other environmental remediation/decontamination treatments. AOPs have gained great importance as alternative treatment processes that affect the degradation of organic species through the action of the hydroxyl radical (OH), oxidizing pollutants present in wastewater and industrial effluents [4]. AOPs are carried out at room temperature and at a pressure close to normal, which involve the formation of very reactive radical species with a high oxidizing capacity, mainly hydroxyl (OH) radicals. These OH radicals are extremely reactive oxidizers (oxidation potential of the OH radical is approximately, E^θ^ = 2.8 V) and non-selective towards organic pollutants in wastewater [5]. AOPs can be considered versatile technologies, as they provide different possible alternatives to produce OH radicals [6]. AOPs, compared to conventional water treatment techniques, have a greater efficiency and capacity to degrade recalcitrant organic pollutants, and can generate less toxic intermediate products during their degradation [7].

Over the last decades, different advanced homogeneous and heterogeneous oxidation processes have been investigated in the field of wastewater treatment. Different mechanisms can be used for the activation of AOPs: ultraviolet radiation (UV) or visible light; different oxidants species (O_2_, H_2_O_2_, O_3_); or catalysts materials (TiO_2_) [8]. The different techniques of AOPs which are currently being used for the treatment of wastewater can be essentially divided into two large groups, the photochemical methods and the non-photochemical methods. Non-photochemical methods do not require light energy to form OH radicals and these methods include ozonization (O_3_), ozonization combined with hydrogen peroxide (O_3_/H_2_O_2_), catalytic ozonization (O_3_/catalysts), Fenton system (Fe^2+^/H_2_O_2_), and ultrasound, among others. With respect to photochemical methods, complete destruction of organic compounds can be achieved by combining UV radiation with non-photochemical AOPs. UV radiation, coming from most UV lamps, has a wavelength between 200 and 300 nm. Photochemical methods include ozonization combined with ultraviolet radiation (O_3_/UV), ozonization with hydrogen peroxide and ultraviolet radiation (O_3_/H_2_O_2_/UV), the photo-Fenton system (Fe^2+^/H_2_O_2_/UV), and photocatalysis, among others [9,10].

Currently, there are several environmental impacts associated with the different conventional technologies in water treatment, and these technologies are sometimes considered inefficient in removing emerging contaminants and endocrine disrupting compounds. Although there are some effective current technologies for water treatment, most of the time, they are technologies that do not always offer the most economical solution for the removal of various pollutants, in particular, for pollutants present in low concentrations, requiring a large amount of energy and producing a considerable amount of hazardous waste [11]. The use of nanomaterials, such as nanoparticles, nanomembranes and nanotubes, have proved to be quite effective for the detection and removal of various chemical and biological substances that include metals, algae, organic substances, bacteria, viruses, nutrients and antibiotics [10]. Nanotechnology describes the characterization, fabrication and manipulation of structures, devices or materials that have one or more dimensions smaller than 100 nanometers [12].

Nanotechnology has been shown to be a promising alternative in water treatment and environmental remediation. Several researches have been carried out in the scope of developing advanced oxidation processes (AOPs) combined with nanotechnology for water treatment. One of the great advantages of using nanotechnology in wastewater treatment is the fact that few by-products that are toxic to the environment are formed. Techniques based on nanotechnology have proved to be extremely important to meet increasingly demanding water quality standards, namely in the removal of emerging pollutants and for lower levels of contaminants [7].

Although the costs associated with nanotechnology are generally considered high, in some cases they can offer more effective alternatives at lower costs than conventional techniques. In addition, the costs of technologies based on nanotechnology may be reduced by production on an industrial scale and from the development of new methods that use cheaper raw materials and consume less energy. Technologies based on nanotechnology also allow the reuse of many nanoparticles, reducing energy consumption, increasing the efficiency of treatment and also reducing the production of associated waste [3,11].

Currently, nanomaterials that have proved important for the degradation of organic matter in wastewater are classified as dendritic polymers, metal/metal oxide nanoparticles, zeolites and carbon-based nanomaterials [13]. Dendritic polymers are nanostructures constituted by the presence of multi-branched chains. Different dendrimer structures can be produced through the reaction between dendritic polymers that consist of multifunctional properties. These dendritic polymers can be used in the process of removing organic pollutants and heavy metals, acting as adsorbents. The characteristics of these dendritic polymers are therefore important for water treatment [10]. Metal/metal oxide nanoparticles are a diverse class of nanomaterials composed of one, two or three metals and/or metal oxides. Silver, gold and palladium nanoparticles are widely studied for wastewater treatment. Metal oxide nanoparticles, such as TiO_2_, ZnO and CeO_2_, stand out in the degradation of organic pollutants in aqueous media, due to their high surface contact area and improved photolytic properties, thus being considered the best photocatalysts for water treatment [11]. Zeolites have a porous structure making them effective in ion exchange, and are therefore widely used as an ion exchange medium for water treatment and also effective in removing metal ions present in wastewater [14].

Carbon-based nanomaterials are composed essentially of carbon atoms, have an exceptionally high surface area, thus making them ideal for the adsorption of pollutants and therefore very useful in the treatment of emerging contaminants, considered an economical option for removing pharmaceutical and personal care products and endocrine disrupting compounds. These nanomaterials have a relatively high removal capacity in organic compounds, making them a very promising option, and the cost is not much higher than other water treatment methods [13].

The unique properties of nanomaterials when combined with AOPs present unique opportunities to revolutionize water treatment. These combined methods of nanomaterials involving AOPs are one of the most advanced techniques for removing contaminants from wastewater or converting pollutants into more degradable compounds, being considered a promising alternative to traditional methods of water treatment and environmental remediation [15]. However, most methods of AOPs combined with nanomaterials are still under study, and it is necessary to further investigate and develop these innovative methodologies in order to increase their potential. The use of nanomaterials in wastewater treatment should be done carefully taking into consideration all sustainability indexes, and particularly taking into account that they should not result in damage to the environment [10,11].

## 2. AOPs and Its Mechanisms

In this section a review of the most important AOPs are presented focusing on their mechanisms of action towards the degradation of water contaminants and pollutants.

### 2.1. UV/H_2_O_2_ Processes

The AOP based on the UV/H_2_O_2_ system consists of the combination of ultraviolet radiation with hydrogen peroxide (H_2_O_2_), leading to its photolysis, in order to generate two hydroxyl (OH) radicals [16], as illustrated in following equation [17]:

$$H_{2}O_{2}\overset{\mathrm{hv}}{\to }2\;\mathrm{OH}$$

In addition to direct photolysis, the UV/H_2_O_2_ process can also comprise indirect photolysis, where the compounds react with the OH radical, produced by the photolysis of H_2_O_2_, in order to generate less reactive radicals such as HO_2_, which can react with the H_2_O_2_, generating again the OH radical [16,18], as shown in the following equations:

$$H_{2}O_{2}+\mathrm{OH}\to H_{2}O+{\mathrm{HO}}_{2}$$

$${\mathrm{HO}}_{2}+H_{2}O_{2}\to H_{2}O+O_{2}+\mathrm{OH}$$

The efficiency of OH radical production depends on the ability of hydrogen peroxide to absorb UV radiation, as well as the physical and chemical characteristics of the fluid that will be subjected to the oxidation process. UV absorption by H_2_O_2_ will depend on the wavelength of UV radiation, that is, the shorter the wavelength of UV radiation, the greater the energy absorption by H_2_O_2_, increasing the production potential of the OH radical [17].

An advantage of the UV/H_2_O_2_ system is due to the fact that UV radiation acts as a disinfectant, physically inactivating the microorganisms and simultaneously assisting the peroxide photolysis, generating highly reactive OH radical species. Thus, the UV/H_2_O_2_ system is currently one of the most promising technologies for wastewater treatment [17,19].

### 2.2. Persulfate Based Processes

Recently developed, the persulfate oxidation technology (S_2_O_8_^2−^) is a new AOP, which has been shown to be a very promising alternative for water and wastewater treatment [20,21].

Due to its high oxidizing power (E^θ^ = 2.01 V), persulfate is considered an emerging oxidant in the degradation of pollutants present in water, moreover, persulfate is relatively stable at room temperature and has a non-selective behavior in regarding the degradation of pollutants [22,23]. Persulfate can also be activated to produce the sulfate radical (SO_4_), an even stronger oxidant (E^θ^ = 2.6 V) than S_2_O_8_^2−^ [24].

There are several methods of activating persulfate, in general, the SO_4_ radical can be generated through heat, ultraviolet light, alkali, ultrasound, transition metal ions and activation of metal oxides [21,25].

Compared to the OH radical, the SO_4_ radical has a longer shelf life, has a wider pH range of action, proved to be more stable and effective in oxidation for water decontamination [26,27]. Furthermore, some studies suggest that after the generation of the SO_4_ radical, it may react with several species in solution to form other active species, such as the OH radical, which will play a key role in the pollutant degradation process [24,27].

### 2.3. Chlorine and NH_2_Cl Based Processes

Chlorine is one of the most used chemical oxidants worldwide for the disinfection of drinking water [28]. Currently, the UV/chlorine process is considered an emerging AOP, constituting an alternative to the UV/H_2_O_2_ process in water treatment, effective in the degradation of a variety of persistent contaminants, such as desethylatrazine, sulfamethoxazole, carbamazepine, diclofenac, benzotriazole, tolyltriazole, iopamidol, 17α-ethinylestradiol [29,30]. Compared to the UV/H_2_O_2_ process, the UV/chlorine process demonstrated greater efficiency in the degradation of some micropollutants under slightly acidic conditions, such as trichloroethylene [31]. In the UV/chlorine process, reactive species such as the OH radical and the Cl radical are formed from photolysis of free chlorine (HOCl/OCl-), as shown in the following equations [30]:

$$\mathrm{HOCL}\overset{\mathrm{hv}}{\to }\mathrm{HO}\cdot +\mathrm{Cl}$$

$${\mathrm{OCL}}^{-}\overset{\mathrm{hv}}{\to }O^{-}+\mathrm{Cl}$$

The Cl formed reacts with chloride, giving rise to Cl_2_^•−^, and both Cl and Cl_2_^•−^ are strong oxidizers with redox potentials of 2.4 and 2.0 V, respectively. The various reactive species, including the OH radical, formed during this process make the UV/chlorine process a promising AOP for controlling a variety of contaminants in water treatment [29]. Furthermore, chlorine that does not react after the UV/chlorine process can provide residual protection in water distribution systems [32].

Recently, photolysis of monochloramine (NH_2_Cl) has also attracted significant interest as a new AOP for the degradation of emerging water contaminants, such as carbamazepine, and for efficiently controlling the formation of disinfection by-products [28]. Furthermore, NH_2_Cl is considered adequate to provide residual disinfection throughout the water distribution system due to its high stability [33].

NH_2_Cl photolysis generates NH_2_ and Cl radicals, and due to their strong interaction with water, significant amounts of Cl radical are converted into OH radicals. As discussed previously the OH radical is a strong non-selective oxidizer against the different pollutants present in water. In contrast, Cl radical is a relatively selective oxidant that reacts with most organic micropollutants. In relation to the NH_2_ radical, there is still not much information about its reactivity to pollutants present in water [34]. Primary radicals can further react with water co-solutes to form secondary radicals (such as CO_3_^•-^ and Cl_2_^•-^). Although knowledge is still quite limited, the UV/NH_2_Cl process demonstrates considerable potential as a new AOP for water treatment [28,34].

### 2.4. Fenton Processes

The Fenton process consists of the formation of OH radicals in an acidic medium, from the decomposition of hydrogen peroxide by the action of an iron catalyst [7]. The Fenton process is a very promising AOP due to the high mineralization promoted under normal conditions of both temperature and pressure, very effective in destroying refractory and toxic organic pollutants present in wastewater [35,36]. The main reactions involved in Fenton processes are [37]:

$${\mathrm{Fe}}^{2+}+H_{2}O_{2}\to {\;\mathrm{Fe}}^{3+}+\mathrm{OH}+{\mathrm{OH}}^{-}$$

$$\mathrm{OH}+H_{2}O_{2}\to {\;\mathrm{HO}}_{2}+H_{2}O^{\;}$$

$${\mathrm{Fe}}^{2+}+\mathrm{OH}\to {\;\mathrm{Fe}}^{3+}+{\mathrm{OH}}^{-}$$

$${\mathrm{Fe}}^{3+}+{\mathrm{HO}}_{2}\to {\;\mathrm{Fe}}^{2+}+O_{2}+H^{+}$$

$$\mathrm{OH}+\mathrm{OH}\to {\;H}_{2}O_{2}$$

Organic pollutant+OH→ Degraded products

The advantage of Fenton processes is due to the fact that they do not require sophisticated equipment or expensive reagents, they are considered ecologically viable processes due to their high performance and their relatively simple approach, which uses less harmful chemicals and cyclical nature, being necessary a lower concentration of these chemicals [37].

A condition of this AOP is the restricted pH range, where the generation of OH radicals during the Fenton reaction is only efficient under acidic pH conditions (with a pH value close to 3) [35,36].

There are different types of Fenton processes, including Fenton, photo-Fenton, electro-Fenton, photo-electro-Fenton, homogeneous and heterogeneous Fenton, among others. Among the various AOPs, it was the Fenton and photo-Fenton processes that proved to be the most effective, energy efficient and least expensive methods for treating recalcitrant compounds, when used exclusively or in conjunction with other conventional and biological methods [37].

### 2.5. Ozone Based Processes

Ozone (O_3_) is a strong oxidizer, which has been widely used in wastewater treatment and drinking water disinfection. Once dissolved in water, O_3_ acts as an oxidant due to its high redox potential (E^θ^ = 2.07 V), leading to the destruction or degradation of organic pollutants, through different pathways, namely molecular ozone (direct) or through the hydroxyl radical (indirect) [38]. The main semi-reactions of ozone in water are [39]:

$$O_{3}+2H^{+}+2e^{-}\to O_{2}+H_{2}O$$

$$O_{3}+H_{2}O+{\;e}^{-}\to O_{2}+2{\mathrm{OH}}^{-}$$

However, direct oxidation of O_3_, in addition to being a relatively slow reaction, is also more selective than indirect reactions [40]. In water, O_3_ undergoes a series of reactions, decomposing into several oxidative species, including the OH radical, which is a stronger oxidant than the original molecular ozone, which reacts with organic and inorganic compounds in a non-selective way, with rates very high reaction rates. Thus, the high oxidative power of ozonization is partially due to the generation of OH radicals [39].

In the presence of other oxidants or irradiation, the yield of the OH radical can be significantly improved, namely through the addition of hydrogen peroxide (O_3_/H_2_O_2_), use of UV irradiation (O_3_/UV), or addition of metallic catalysts, increasing the efficiency of the treatment. These processes are called advanced oxidation processes (AOPs). Thus, ozonation and ozone-based AOPs are responsible for the destruction of many recalcitrant organic compounds in water and wastewater, including pharmaceuticals and personal care products, solvents, surfactants and pesticides [38,39].

### 2.6. Heterogeneous Photocalalytic Processes

Heterogeneous photocatalysis is an AOP that has been widely studied in the last two decades. The principle of heterogeneous photocatalysis is associated with the activation of a semiconductor by the action of light, when the semiconductor and the reagent are in different phases, photocatalytic reactions are classified as heterogeneous photocatalysis [41].

The substrate that absorbs light and acts as a catalyst for chemical reactions is known as a photocatalyst, and all photocatalysts are generally semiconductors. When a photocatalyst is exposed to light of the desired wavelength (sufficient energy), photoexcitation of an electron occurs, which is promoted from the valence band to the conduction band. The absorption of a photon with energy equal to or greater than the separation of the bands (band gap) of the catalyst, thus creates a gap in the valence band. The electron and the gap migrate to the surface of the photocatalytic semiconductor where they act, respectively, as a reducing agent and an oxidizing agent. On the catalyst surface, the adsorbed water molecules are oxidized by the holes, to OH radicals, which can subsequently oxidize the organic matter in solution, transforming it into non-toxic or less harmful products, such as CO_2_ and water [9].

Among the various existing photocatalysts, TiO_2_ is currently the most studied in heterogeneous photocatalysis processes, essentially due to its physical and chemical characteristics. TiO_2_ has high chemical and thermal stability, is non-toxic, inexpensive and has a relatively high efficiency [41].

As a green, highly efficient and ecological technology, heterogeneous photocatalysis for the treatment of organic pollutants present in wastewater has been shown to be a promising technology to face future environmental challenges [42].

## 3. Nanomaterials Based AOPs

In this section the successful coupling of nanomaterials with some AOPs in water treatment are presented and briefly discussed. Table 1 shows some examples of nanomaterials based AOP for the treatment of dyes in water. The analysis of this table shows that the nanomaterials based AOP allow almost complete degradation of the dyes under investigation and faster decomposition processes are achieved when compared with the processes without nanomaterials. Indeed, innovative processes are being proposed for the treatment of dye-contaminated wastewaters with relative reduced costs and with high efficiency [43].

### 3.1. UV/H_2_O_2_ Processes

The UV/H_2_O_2_ process is a promising technology for the degradation of a wide spectrum of organic contaminants present in water. The coupling of nanomaterials to this process has great potential for water treatment, as the extremely small size of these particles maximizes the surface area exposed to the reagent, allowing more reactions to occur, thus increasing the rate of degradation of these contaminants [3].

The UV/H_2_O_2_ process, by itself, cannot efficiently degrade target pollutants, since the molar absorption coefficient of H_2_O_2_ is relatively weak in the UV region [3]. It will be beneficial if this process coupled with nanomaterials, among which TiO_2_ is the most common. TiO_2_ is a semiconductor that exhibits a wide band interval (3.2 eV) corresponding to radiation in the range close to UV. When UV radiation is irradiated on the TiO_2_ surface, a reactive electron-hole pair is generated, which in turn reacts with H_2_O, forming a hydroxyl radical [10,44].

Thus, the combination of TiO_2_ to the UV/H_2_O_2_ process increases the degradation rate, forming more active radicals. The UV/H_2_O_2_/TiO_2_ process thus proves to be quite efficient in removing persistent organic contaminants from water [45,46,47].

Zinc oxide (ZnO) nanoparticles, when combined with the UV/H_2_O_2_ process, have also proven to increase the effectiveness of this process, with a greater production of hydroxyl radicals, which in turn has a positive impact on the removal of target pollutants [48].

**Table 1:** Some examples of nanomaterials based AOP for the treatment of waste water containing dyes.

AOP Process	Dye	System	Kinetic Parameters	Reference
UV (430 nm)/H_2_O_2_		TiO_2_	100% degradation; *k* = 0.017 min^−1^	[46]
	Real textile effluents	Fe^2+^, Fenton	91% degradation; *k* = 0.0033 min^−1^	
		Control	57% degradation	
Fenton and Ozone	Reactive Red 24	Iron slag (IS) + O_3_	*k* = 0.0327 min^−1^ (pH = 11)	[49]
		Ozone Ozone/H_2_O_2_/IS	*k* = 0.0271 min^−1^ (pH = 11) *k* = 0.0434 min^−1^ (pH = 11)	
UV/Ozone	4-nitroaniline	ZnO nanoparticles	96% degradation; *k* = 0.03 min^−1^	[50]
Heterogeneous photocatalytic	Reactive Black 5	TiO_2_–zeolite Control (commercial TiO_2_ particles)	*k* = 0.0419 min^−1^ *k* = 0.0297 min^−1^	[51]
Heterogeneous photocatalytic	Methylene blue Malachite green Direct blue-15 Amaranth	Nano-TiO_2_ (ultraviolet light—4 h)	93% degradation 88% degradation 95% degradation 85% degradation	[52]
Heterogeneous photocatalytic (visible light)	Methyl orange	TiO_2_ nano 1%Co-TiO_2_ nano	16.3% degradation; *k* = 0.00075 min^−1^ 33.3% degradation; *k* = 0.00164 min^−1^	[53]
Heterogeneous photocatalytic	Methylene blue	TiO_2_ nanoparticles/PES nanofibers	95% degradation *k* = 0.0147 min^−1^	[54]
Heterogeneous photocatalytic	Methylene blue	Titanate nanotubes/Graphite Oxide	97.5% degradation *k* = 0.02845 min^−1^ (t = 25 °C)	[55]

### 3.2. Persulfate Based Processes

Recently, as an alternative to hydroxyl radicals, sulfate radicals have proven to be very effective in removing organic pollutants from aqueous solutions. For the generation of these sulfate radicals, several nanomaterials have been studied in order to make this process more efficient [3,26].

Magnetic iron oxide nanoparticles (MNPs) are a promising technology for the degradation of recalcitrant organic contaminants, such as p-nitroaniline (PNA), in wastewater, as these nanoparticles can effectively activate persulfate, generating sulphate free radicals, due to their relatively wide availability and their specific structural, magnetic and catalytic properties [56]. Furthermore, the excellent ferromagnetic behavior of Fe_3_O_4_ makes it a bifunctional material that can be easily separated from the solution. Thus, the use of these nanoparticles as catalysts in persulfate activation demonstrates a high potential in wastewater treatment [26].

In addition to magnetic iron oxide nanoparticles (MNPs), other nanomaterials have also been proposed as promising heterogeneous catalysts in persulfate activation, due to their high efficiency and good dispersion. Namely, nanomaterials such as ferrite-carbon aerogel, cobalt, iron, Co_3_O_4_/graphene oxide, CoFe_2_O_4_/titanate nanotubes, Co–MnO_4_, α-MnO_2_, have been used for the generation of sulfate radicals, with very interesting results [3].

### 3.3. Fenton Processes

The Fenton process is one of the most efficient processes for removing toxic organic pollutants present in wastewater. Heterogeneous solid catalysts have been widely studied for Fenton-type reactions, as they are more beneficial when compared to homogeneous analogues, due to their wide pH range and easy separation and recovery of these catalysts [3,57].

Fenton-type reactions using nano-zero-valent iron (NZVI) have recently emerged as a type of heterogeneous Fenton reaction, quite promising in wastewater treatment and groundwater remediation. NZVI is able to effectively transform, degrade and remove hazardous contaminants from water [58,59].

Due to its structure, NZVI has several benefits, such as high activity, non-toxicity and reduced cost. The main advantage of NZVI is its high reactivity and efficiency attributed to its particle size at the nanometer scale and its high specific surface area, which provides excellent properties for removing contaminants in aqueous solutions [59]. NZVI nanoparticles can also be combined with metals such as Cu and Pd or metal oxides such as ceria, acting as efficient catalysts in the degradation of target pollutants [3].

Recently, a wide range of nanomaterials has been studied, acting as catalysts in Fenton-type processes, such as iron nanoparticles, carbon nanocomposites, solid iron oxide, activated carbon and Fe_3_O_4_ magnetic nanoparticles. These nanomaterials showed significant results, due to their high surface areas and porosity, which results in a greater production of hydroxyl radicals, increasing the efficiency of Fenton processes in water treatment [3,57].

### 3.4. Ozone Based Processes

Ozone is considered to be one of the most powerful and favorable oxidants in eliminating toxic organic compounds. However, by itself, ozone has a slow reaction rate towards some persistent organic compounds, such as inactivated aromatics. Thus, currently, some catalysts have been used to increase the oxidation of persistent organic compounds in wastewater, based on catalytic ozonization processes [49,60].

Recently, some nanomaterials were applied as heterogeneous catalysts, to increase the yield of the O_3_/H_2_O_2_ process, among them metallic slags stand out. Iron slag emerges as an excellent catalyst due to their availability and reduced cost [59]. The use of these nanomaterials and nano-zero-valent iron in wastewater treatment will remarkably reduce the overall cost of wastewater treatment [3,49].

ZnO and TiO_2_ nanoparticles were studied as heterogeneous catalysts for the O_3_/UV process, where promising results were obtained. These nanoparticles were considered excellent catalysts in the generation of oxidizing species in the presence of photons [3]. ZnO has attracted special attention due to its high sensitivity to ultraviolet light, its non-chemical nature, high stability, high surface-to-volume ratio, long service life, and high efficiency in the production of electrons. Thus, the UV/ZnO/O_3_ process is currently considered an ecologically correct method to treat large volumes of wastewater containing toxic and resistant pollutants [50].

There is also a wide range of nanomaterials, which have been studied as heterogeneous catalysts in ozonation, in order to improve their reactivity with different types of organic pollutants. For example, NiFe_2_O_4_, Co_3_O_4_, Fe_3_O_4_, MgO, Mn/γ-Al_2_O_3_ and Fe_3_O_4_/carbon nanotubes, which obtained very interesting results [3].

### 3.5. Heterogeneous Photocalalytic Processes

Heterogeneous photocatalysis is an emerging branch of AOPs for water treatment. Nanometric sized photocatalysts are preferentially used in heterogeneous photocatalysis due to their high specific surface area, which gives them greater profitability in the degradation of persistent pollutants in water [51].

Currently, TiO_2_ nanoparticles have been widely applied in photocatalysis for organic reactions and degradation of organic pollutants, due to their specific properties, such as their strong oxidative power, high chemical and thermal stability, low toxicity, reduced cost and availability in large quantities [61]. Furthermore, the ability of TiO_2_ nanoparticles to degrade organic compounds also depends on the particle size, as these particles have a nanometric size, which gives them larger specific surface areas. TiO_2_ is thus the most promising photocatalyst for the degradation of contaminants present in water [52].

Recently, the combination of some titanium nanoparticles with other compounds was also investigated, namely, with carbon spheres and nanotubes, zeolites, graphene oxide, mp-MXene/TiO_2−x_ and Au-TiO_2_/SiO_2_ nanodots, with the aim of to increase the efficiency of heterogeneous photocatalytic processes in the degradation of target contaminants [3,62].

In addition to TiO_2_, there are also other semiconductors that have also been successfully used in the photocatalytic degradation of pollutants, such as zinc oxide (ZnO), this is the second most studied photocatalyst for the photocatalytic degradation of target pollutants. The main advantage of ZnO is that it absorbs a fraction of the solar spectrum greater than TiO_2_, therefore, the ZnO photocatalyst is sometimes considered more suitable for photocatalytic degradation of refractory organics in the presence of sunlight [3,63].

The use of anatase TiO_2_ nanoparticles as a heterogeneous catalyst in water treatment has one drawback related with its wide band gap of 3.2 eV, that limits its workability under UV light—anatase TiO_2_ absorb only wavelengths lower to about 387 nm [64]. To overcome this constraint and make TiO_2_-based nanomaterials photosensitive under visible light, doping TiO_2_ with metal and/or non-metal ions is being reported [53,65,66]. This strategy results in an overall (UV and visible light) photocatalytic enhancement in consequence of the suppression of the electron/hole recombination in anatase [67,68,69].

## 4. Nanomaterials as Adsorbents in Water Treatment

In this section a brief global description is presented of nanomaterials designed to be selective adsorbents of contaminants that exist in fresh water and wastewater. A process unit of adsorption of selective undesirable substances in the water should not constitute per se an AOP. In order for the above AOPs based nanomaterials to be effective in a degradable substance elimination, an elementary step of adsorption/absorption of the substance to the nanoparticle may exist, which facilitates the catalytic process due to the proximity to the catalyst or due to a synergistic mechanistic effect between the adsorbent and catalytic nanomaterials. This can be achieved by using nanocomposite materials constituted by an adsorbent and a catalyst, which may be particularly useful for organic substances degradation, such as dyes [70]. Indeed, the removal of dyes from water can be achieved by adsorption and/or degradation of the organic molecules.

The coupling of TiO_2_ with an intrinsically adsorbent material, polyethersulfone nanofibers (PES NF), resulted in an efficient adsorption-photocatalytic degradation nanosystem for methylene blue dye [54]. Graphene oxide (GO), a common adsorbent nanomaterial with similar properties to that of graphene, was grafted to titanate nanotubes (TNT@GO), that are produced from the alkaline and thermal treatment of TiO_2_ and showed higher performance in the photocatalytic activity for degrading MB dye [55]. GO is characterized by a high adsorption capacity and stability and is a particularly interesting nanomaterial to be coupled to photocatalytic nanomaterials, such as for example TiO_2_ and derivatives, because it shows some semiconductive properties that can serve as photosensitizer via suppressing the charge recombination rate of electron-hole pairs [19].

The removal of metal ions soluble in water can be achieved by adsorption to the solid phase of the nanomaterial [71,72,73,74]. Additionally, some water remediation can be foreseen by the transformation, oxidation or reduction, of a metal ion into a less toxic species. A huge number of nanomaterials have been developed as metal ion adsorbents and the most important classes are [71,72,73,74]:

(i). Carbon nanomaterials: carbon nanotubes, graphene based materials, graphitic carbon nitride, nanoporous carbon, carbon nanofibers, fullerene, nanodiamonds;
(ii). Metal and metal oxides: NZVI, Al_2_O_3_, silica, Fe_3_O_4_, MnO_2_, TiO_2_, MgO, CdO, ZnO;
(iii). Magnetic nanoparticles functionalized with; biomolecules (for example, alginate and cellulose), organic molecules, carbonaceous materials, inorganic molecules (for example, EDTA);
(iv). Polymer-based nanomaterials: chitosan, dendrimers, cellulose, zeolite.

## 5. Perspectives

The current worldwide water crisis will get increasingly worst in the next decades. A critical part of the current and future water management will be the water treatment of wastewater, with particularly relevance to the industrial effluents, and drinking water. Wastewater regulatory frameworks will be increasingly strict to reduce environmental impacts and to allow water to be reused. Drinking water quality standards must be improved in order to account for the emerging pollutants that are being detected in tap water. These tasks can only be achieved if new improved and sustainable water treatment technologies are developed.

Nanomaterials will definitely be part of the solution for future water managing strategies. Indeed, nanomaterials will enhance the performance of the current technologies sustainably, because fewer resources are used in their production and greener catalytic processes are implemented. Moreover, when compared with bulk materials, nanomaterials have unique properties that allow the development of new and innovative AOP. Consequently, the potential for the incorporation of nanomaterials in high environmental performance AOPs is enormous and will continue to occur in the near future.

New nanomaterials development is necessary for the water treatment industry. Nanomaterials with a multifunctional role are most desirable, for example with adsorption, radiation active properties (UV and visible light), and reactive oxygen species generation, etc. However, multifunctional advanced innovative processes can also be developed by the coupling of nanomaterials with bulk materials resulting in tuned systems that can be used in water treatment.
